# Variability in hemoglobin levels in hemodialysis patients in the current era: a retrospective cohort study 

**DOI:** 10.5414/CN109031

**Published:** 2017-09-07

**Authors:** David T. Gilbertson, Yan Hu, Yi Peng, Bradley J. Maroni, James B. Wetmore

**Affiliations:** 1 Chronic Disease Research Group, Minneapolis Medical Research Foundation, Minneapolis, MN, and; 2 Akebia Therapeutics, Inc., Cambridge, MA, USA

**Keywords:** anemia, hemodialysis, hemoglobin variability, mortality, major adverse cardiac events

## Abstract

Background: Given regulatory and reimbursement changes in anemia management, we examined hemoglobin variability in a contemporary cohort of maintenance hemodialysis patients. Materials and methods: The study population included > 200,000 hemodialysis patients with Medicare parts A and B as primary payer on October 1, 2012. Based on 25^th^ and 75^th^ percentiles, monthly hemoglobin values were categorized as low, intermediate, or high. Six variability categories were created by patterns during the 6-month observation period. Stable categories were: always-low, always-intermediate, always-high; variable patterns were: varying between low and intermediate, intermediate and high, low and high (most-variable). Cox proportional hazard models were used to assess the association between hemoglobin variability and all-cause mortality or major adverse cardiac events (MACE). Results: The 25^th^ and 75^th^ hemoglobin percentiles were 10.2 and 11.5 g/dL, respectively, in 2012, vs. 11 and 12.5 g/dL in 2004. ESA doses were lower in all categories in 2012 and transfusion rates higher, particularly for always-low patients. Hemoglobin variability decreased modestly: in 2004, 6.0% were always-intermediate, vs. 9.5% in 2012. In 2012, more patients were always-high and fewer were most-variable. Mortality hazard ratios (HRs) were higher for patients with any low hemoglobin: always-low (HR, 95% CI: 2.07, 1.84 – 2.31), varying between low and intermediate (1.37, 1.29 – 1.45), and most-variable (1.23, 1.16 – 1.31); the pattern was similar for MACE. Conclusions: In 2012 vs. 2004, hemoglobin levels decreased, the range of levels narrowed, and variability decreased modestly; transfusions increased. The highest risk of mortality and MACE appeared to occur in patients with persistently low, rather than highly variable, hemoglobin levels.

Supplemental material is available for free at: http://www.clinnephrol.com Vol. 88 – November 2017

## Introduction 

The hypothesis that hemoglobin variability, or within-patient changes in hemoglobin levels over time, is associated with adverse outcomes in patients receiving maintenance dialysis was posited over a decade ago. An early seminal study conducted by Berns et al. [1] first characterized the phenomenon of hemoglobin variability. Many studies followed [2, 3, 4, 5, 6, 7, 8, 9] characterizing hemoglobin variability in different ways while relating it to patient characteristics and to outcomes in maintenance hemodialysis patients, nondialysis chronic kidney disease patients, and kidney transplant recipients. These studies showed that variability was ubiquitous and generally associated with a higher comorbidity burden and more frequent hospitalizations. Subsequent studies [10, 11, 12, 13, 14, 15, 16, 17, 18, 19, 20] showed an association of hemoglobin variability with mortality, although some suggested that residual confounding, not variability per se, partially explained the association, since patients with unstable health status are at risk for hemoglobin fluctuations. Further work [17] suggested that lower hemoglobin levels (most likely due to intercurrent illnesses and hospitalizations), rather than hemoglobin variability per se, were associated with adverse outcomes. 

Most of these analyses were conducted during an era of increasing hemoglobin levels, so findings must be contextualized within that clinical and regulatory environment. Hemoglobin levels peaked in 2007, then began to fall after publication of several studies that implicated high hemoglobin levels in cardiovascular events [21, 22, 23]. Hemoglobin levels decreased steadily, a trend accelerated by the introduction of the revised Prospective Payment System (PPS) by the Centers for Medicare & Medicaid Services (CMS) in January 2011. Designed in part to control costs associated with erythropoiesis-stimulating agents (ESAs) [24], the implementation of the PPS, along with other broadly contemporaneous events such as the Quality Improvement Program implemented by CMS [25], the ESA label change by the US Food and Drug Administration [26], and revised anemia management clinical practice guidelines [27, 28, 29], was associated with a decrease in mean hemoglobin levels from 11.3 g/dL in 2011 to 10.5 g/dL by late 2013. 

Given this substantial change in average hemoglobin levels due to newer anemia management guidelines [25, 26, 27] and changes in the reimbursement environment, we sought to examine hemoglobin variability in a contemporary cohort of patients receiving maintenance hemodialysis to ascertain patient characteristics associated with variability and to determine whether variability was associated with adverse outcomes. We hypothesized that, while changing anemia management practices have led to a decline in average hemoglobin levels, variability remains and is associated with all-cause mortality and the composite of all-cause mortality, nonfatal stroke, and nonfatal myocardial infarction (i.e., major adverse cardiac events (MACE)). 

## Materials and methods 

### Development and characterization of the cohort 

We used previously employed methodology to permit comparison between the results of the current study and previous work [4, 6]. The study population consisted of maintenance hemodialysis patients with Medicare Parts A and B as primary payer as of October 1, 2012. A Medicare-covered 6-month baseline period before October 1 was used to ascertain hemoglobin variability and comorbid conditions. Medicare Part A (inpatient, long-term care hospital, outpatient, home health, hospice, and skilled nursing facility) and Part B (physician/supplier) claims, and the Medical Evidence Report (form CMS-2728) were used to ascertain comorbidity. Presence of comorbidity was established, as has been done previously [21, 22], by the presence of at least 1 inpatient hospitalization, skilled nursing facility, or home health agency code or at least 2 outpatient or physician/supplier codes, or at least 1 outpatient code and at least 1 physician/supplier code, on different dates less than 1 year apart. Comorbid conditions examined were diabetes, atherosclerotic heart disease, congestive heart failure, dysrhythmia, other cardiac disease, cerebrovascular event or transient ischemic attack, peripheral arterial disease, chronic obstructive pulmonary disease (COPD), gastrointestinal bleeding, liver disease, and cancer. Relevant International Classification of Diseases, Ninth Revision, Clinical Modification (ICD-9-CM) codes appear in Supplemental Table S1. Information on recombinant erythropoietin (epoetin-α) and intravenous (IV) iron use was obtained from dialysis facility claims, and on red blood cell transfusions from inpatient, outpatient, skilled nursing facility, and physician/supplier claims. 6-month (primary) and 1-year (sensitivity) follow-up periods beginning October 1, 2012, were used to assess outcomes. Supplemental Figure S1 presents a timeline showing the baseline and follow-up periods. 

### Categorization of hemoglobin levels 

Consistent with previous work by our group [7], monthly hemoglobin values were categorized as low (L), intermediate (I), or high (H), where L and H were based on monthly values below or above the 25^th^ and 75^th^ percentiles, respectively. Hemoglobin variability was then classified into six groups based on the lowest and highest categories during the 6-month baseline observation period (LL, consistently low; II, consistently intermediate; HH, consistently high; LI, low-intermediate; IH, intermediate-high; LH, low-high). Epoetin-α use was determined by the number of months during the 6-month baseline period in which at least 1 dose of epoetin-α was received, and by average monthly dose for patients receiving epoetin-α. IV iron use was characterized by the number of months in which at least 1 dose of any IV iron product was received, and for patients who received IV iron, by the average monthly dose of iron sucrose or sodium ferric gluconate. Transfusions were evaluated based on the percentage of patients who received at least 1 transfusion during the baseline period, and by the monthly percentage of patients who received at least 1 transfusion. Hospitalizations were determined by the percentage of patients who were hospitalized at least once during the 6-month baseline period, and by total hospitalization days. 

### Outcomes 

Two endpoints were analyzed during the 6-month and 1-year follow-up periods: all-cause mortality, and a composite of MACE comprising all-cause mortality, nonfatal stroke, or nonfatal myocardial infarction (MI). Mortality information was obtained from the Death Notification (form CMS-2746). The combined endpoint was ascertained from inpatient MI or stroke claims with qualifying ICD-9-CM diagnosis codes in any position. MI was identified using diagnosis codes 410.x0 or 410.x1 and stroke using codes 430.x, 431.x, 434.x or 436.x. All-cause mortality for the 2012 cohort was contrasted with previously reported results from 2004; comparison of MACE events between the two time periods could not be conducted, since this outcome was not measured in the earlier study [7]. 

### Statistical analysis 

Means and standard deviations were reported for continuous variables and percentages for categorical variables. Cox proportional hazard models were used to assess the association between hemoglobin variability group and the outcomes all-cause mortality or MACE within 6 or 12 months, first unadjusted and then adjusting for patient demographics, cause of renal failure, time on dialysis, and comorbidity. The proportional hazards assumption was checked for each model by creating time-varying interaction terms of each variable and follow-up time. All analyses were conducted in SAS, version 9.3 (Cary, NC, USA). The study was approved by the Institutional Review Board of Hennepin County Medical Center, Minneapolis, MN, USA. 

## Results 

Table 1 shows patient characteristics overall and by variability group for the 2004 and 2012 data. Compared with the 2004 cohort, the 2012 cohort was slightly older, more likely to be male, and generally similar with respect to race and cause of renal failure. Total hospital days were fewer in 2012, consistent with declining hospitalization rates over the past decade in both the general and dialysis populations. Comorbid conditions were generally similar, but diabetes, dysrhythmias, and COPD were slightly more common in 2012. Patient characteristics across variability groups showed similar patterns in the 2004 and 2012 cohorts; for example, patients in the LL groups in both cohorts were younger and more likely to be black, to have renal failure due to causes other than diabetes or hypertension, and to have more hospital days and comorbid conditions. Patients in the II, HH, and IH groups had fewer hospital days and less comorbidity than those with lower hemoglobin levels. 

**Figure 1.**
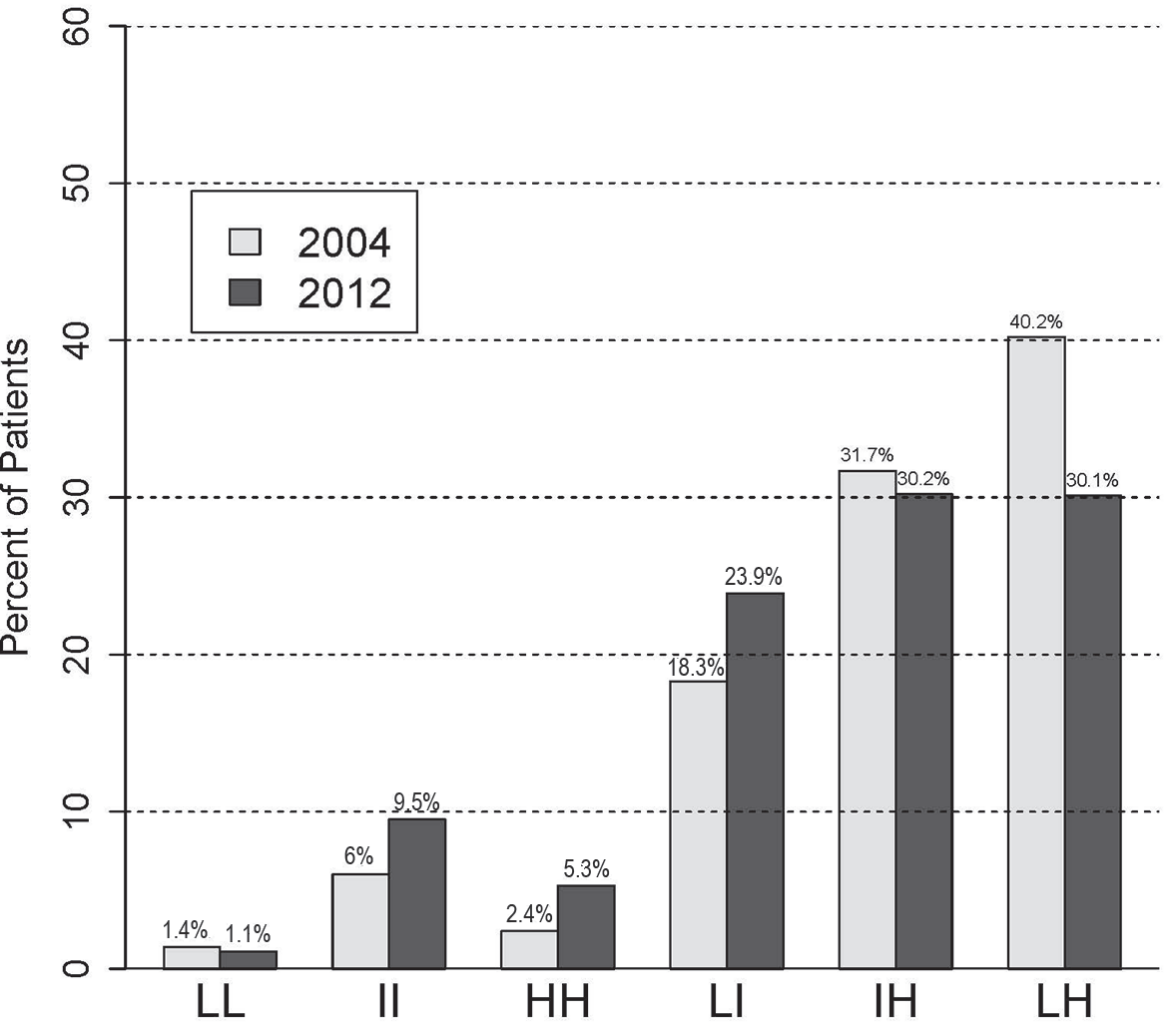
Proportions of patients in each hemoglobin variability group, 2004 and 2012 cohorts. LL = consistently low; LI = low-intermediate; LH = low-high; HH = consistently high; IH = intermediate-high; II = consistently intermediate.

In 2012, the 25^th^ and 75^th^ hemoglobin percentiles were 10.2 and 11.5 g/dL, respectively. Analogous values for the 2004 cohort were 11 and 12.5 g/dL. The distribution of the hemoglobin variability groups for both cohorts is shown in Figure 1, which generally demonstrates less hemoglobin variability in 2012 than in 2004. For example, 9.5% of the 2012 cohort were in the most stable (II) group, compared with only 6.0% of the 2004 cohort, while the percentage with the most pronounced variability (the LH group) decreased from 40.2% in 2004 to 30.1%. 

**Figure 2.**
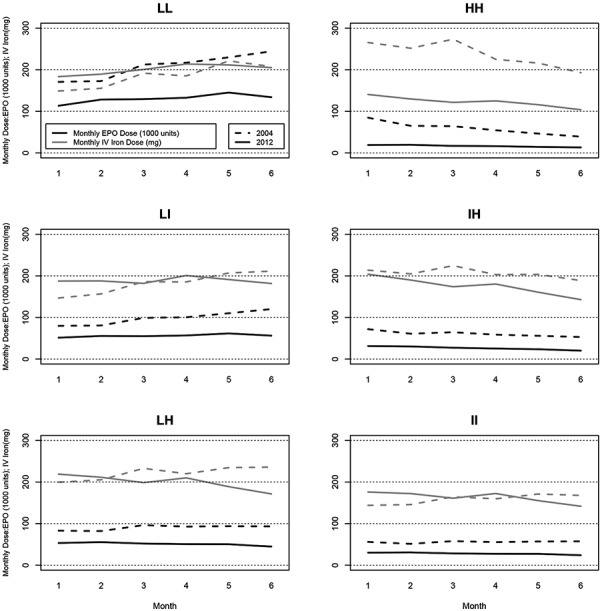
Monthly epoetin-α dose (black lines) and IV iron dose (gray lines) by hemoglobin variability group. Dotted lines are 2004, solid lines are 2012. Legend in top left panel applies to all panels in the figure. LL = consistently low; LI = low-intermediate; LH = low-high; HH = consistently high; IH = intermediate-high; II = consistently intermediate.

Epoetin-α doses for each hemoglobin variability group are shown for the 2012 (black solid lines) cohort for purposes of comparison with the 2004 group (black dotted lines) in Figure 2. Consistent with generally lower overall epoetin-α doses since 2011, epoetin-α use was lower in 2012 than in 2004 for each hemoglobin variability group. Most strikingly, the 2012 LL group showed smaller increases over time than the 2004 LL group, which showed consistently increasing monthly epoetin-α doses over the baseline period. The pattern for the LI group was similar but less marked. While the 2004 HH group showed consistently decreasing monthly epoetin-α doses, doses in the 2012 HH group were relatively unchanged over the baseline period. 

Monthly IV iron doses are also shown in Figure 2 (gray lines). Doses in the HH group were much lower in 2012 than in 2004, although doses tended to decrease in both cohorts over 6 months. Dose decreases in the LH and IH groups were more marked in the 2012 cohort than in the 2004 cohort. While the II group showed relatively consistent or slightly increasing doses in 2004, doses decreased for this group in 2012. 

Transfusion use in the LL group was higher in 2012 (mean percentage transfused, 19.9%) than in 2004 (mean percentage transfused, 16.5%), but, unlike in 2004, did not show a pattern of month-to-month increases (not shown). Transfusions were also slightly more common in 2012 than in 2004 in other variability categories with at least 1 low hemoglobin month (i.e., the LI and LH groups). 

**Figure 3.**
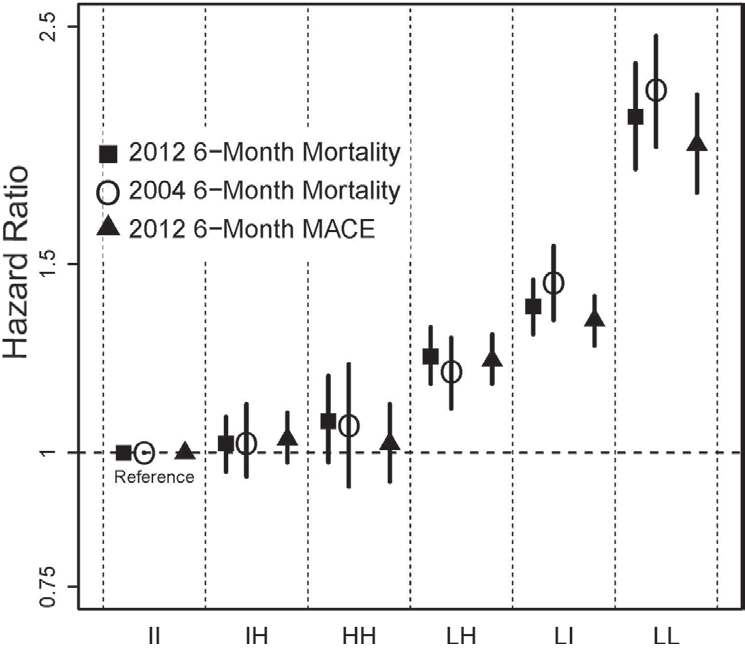
Adjusted association between hemoglobin variability and 6-month outcomes, 2004 and 2012 cohorts. LL = consistently low; LI = low-intermediate; LH = low-high; HH = consistently high; IH = intermediate-high; II = consistently intermediate; MACE = major adverse cardiac events.

The association between hemoglobin variability and outcomes (i.e., mortality and MACE in 2012, mortality in 2004) during the 6-month follow-up period is shown in Figure 3; analogous results during the 12-month follow-up period are shown in Supplemental Figure S2. Supplemental Figures S3 and S4 show unadjusted and adjusted results for 6-month mortality and 6-month MACE, thereby demonstrating the effect of comorbidity and demographic adjustment. Full model results for the 6-month follow-up period for the 2012 cohort are shown in Supplemental Table S2. Hazard ratios (HRs) for all outcomes for patients with low hemoglobin levels at any point were higher than for those whose levels were never low, and HRs for patients with consistently low levels (the LL groups) were substantially higher than for all other groups. For example, for the LL group, the HR (95% CIs) for 6-month mortality was 2.07 (1.84 – 2.31) and for 6-month MACE 1.95 (1.75 – 2.17), compared with the referent (II) group. For the LI group, corresponding HRs decreased to 1.37 (1.29 – 1.45) for mortality and 1.33 (1.26 – 1.40) for MACE. For the LH group, HRs decreased further still, to 1.23 (1.16 – 1.31) for mortality and 1.22 (1.16 – 1.29) for MACE. Generally, HRs for mortality in each variability group were similar in the 2012 and 2004 cohorts. Comparing unadjusted with adjusted results (Supplemental Figures S3 and S4), adjustment generally attenuated HRs toward 1, as might be expected, albeit modestly. The tests of the proportional hazards assumption showed no violations of the assumption. 

## Discussion 

In this study, we sought to ascertain the degree of hemoglobin variability in contemporary hemodialysis patients, and whether hemoglobin variability has changed since the introduction of the revised PPS, implementation of the Quality Improvement Program by CMS [25], and the ESA label change by the US Food and Drug Administration. Additionally, we examined how anemia management differs by degree of hemoglobin variability and how hemoglobin variability is associated with adverse outcomes. Consistent with other reports [30, 31], we found that mean hemoglobin levels have decreased by ~ 1 g/dL since 2004. We also found that, despite somewhat less variability in 2012 than in 2004, hemoglobin management remained a substantial challenge in the care of hemodialysis patients, with almost all patients moving between categories over fairly short time periods. Despite less variability, transfusions were administered more frequently in 2012 than in 2004, not only in patients with consistently low hemoglobin levels (LL), but also in those whose levels were ever low (LI and LH). We also found that low hemoglobin levels, rather than hemoglobin variability itself, appeared to be most strongly associated with the highest risk of mortality and MACE. 

Hemoglobin variability in hemodialysis patients in the current era of anemia management has not, to our knowledge, been previously explored. In addition to reaffirming previous reports showing a mean hemoglobin decrease of ~ 1 g/dL, we demonstrate that the changes in hemoglobin levels appear to represent a population-wide “frame shift,” since hemoglobin levels in patients at the 25^th^ and 75^th^ percentiles also dropped by similar amounts. Hemoglobin variability appeared to be somewhat less in 2012 than in 2004. For example, a slightly higher percentage of patients had consistently intermediate (II) values, while a lower percentage was in the most variable group (LH). Reasons for this are uncertain, but it could be due to widespread implementation and refinement of anemia management protocols by dialysis providers, who in more recent years have scrutinized this clinical issue more than in the past. 

Despite a decrease in the magnitude of hemoglobin level variability overall, however, variability remained ubiquitous. Only 15.9% of all patients were in groups that could be termed “consistent” (II, LL, HH), representing only a modest improvement from 9.8% in 2004. Given that the intermediate category was defined as a hemoglobin level of 10.0 – 11.5 g/dL, it could be argued that levels consistently below 10.0 or above 11.5 g/dL (the LL and HH groups, respectively), do not represent optimal management. As such, substantial hemoglobin variability continues to characterize anemia management in patients receiving maintenance dialysis, and improvements between 2004 and 2012 were modest at best. 

The present study also complements and extends previous work on anemia treatment by considering the relationship between hemoglobin variability and management. As with other studies [32, 33], we demonstrate substantially lower use of epoetin-α after the introduction of the PPS. However, some additional findings are worth noting. Mean epoetin-α dose was most strikingly lower in the LL group in 2012 compared with 2004, suggesting that epoetin-α is not being used as aggressively to manage patients with persistently low hemoglobin levels. This pattern was also evident, to a lesser degree, in the LI and LH groups. In contrast, iron use, which decreased overall in the period studied (most dramatically in the HH group), increased in the LL group, suggesting that iron may have been employed as a partial alternative to epoetin-α in patients with persistently low hemoglobin levels. 

One key finding concerned the relationship between hemoglobin variability and use of red blood cell transfusions. While other studies have demonstrated increasing use of transfusions in recent years [34, 35, 36], the present report provides additional detail. Transfusions increased most strikingly in the LL group, suggesting that transfusions have become a more important treatment option in patients with persistently low hemoglobin levels. It is possible that transfusions are now prescribed more readily in LL patients in an environment of declining ESA use – even in patients with low hemoglobin levels – and thus might be increasingly used as “acute” or “rescue” therapy for such patients; whether this represents an optimal treatment strategy warrants further study. Also, more transfusions were administered in the LH group in 2012 than in 2004; the LH group constitutes a much larger number of patients than the LL group, and therefore contributes a greater share to the total transfusions administered. The LH patients likely represent two distinct subgroups, those who receive a transfusion due to an acute decrease in hemoglobin level (that is, move from high to low), and those who receive a transfusion due to a low hemoglobin level and attain a high level. While the former scenario is relatively common given the clinical instability of maintenance hemodialysis patients, the latter represents a group whose management could be further scrutinized, since attainment of high hemoglobin levels might not represent optimal management. Further work should investigate the clinical scenarios represented by these apparently volatile LH patients, and the treatment strategies that might best be applied to them. Whether transfusions could be managed more judiciously – such as being coupled with other therapies, to prevent undesirable rises in hemoglobin levels – justifies further study. 

The present study supports earlier work [17], which found that persistently low hemoglobin levels, rather than hemoglobin variability per se, is associated with adverse outcomes. Patients in the 2012 LL group had a roughly 2-fold greater likelihood of experiencing either death or MACE compared with stable patients with intermediate hemoglobin levels. Risk estimates for the LI and LH groups demonstrated an expected ordinal response. Absolute estimates were comparable to those in 2004, suggesting the continued importance of persistently low hemoglobin levels. Because causality cannot be determined by the present study design, it is uncertain whether low hemoglobin levels contribute to poor outcomes or whether patients who are ill or inflamed are at risk of both low hemoglobin levels and poor outcomes. Given recent work suggesting that rates of mortality, stroke, MI, heart failure, and venous thromboembolic disease have not increased after the introduction of the revised PPS [37], we suspect the latter, but more work is needed in this regard. 

The present study has a number of important limitations. The classification of variability as low, intermediate, and high is arbitrary. However, the cutpoints, designated at the 25^th^ and 75^th^ percentiles, reflect convention, and were selected to permit direct comparison to our previous work [4, 11]. The baseline and follow-up periods selected for assessment of variability and of subsequent outcomes were also arbitrary but consistent within our previous work [4, 11]. The concept of “variability” identified only changes in hemoglobin levels, not directionality; thus, patients whose hemoglobin levels rose were modeled identically to those whose levels fell. Relatedly, whether treatments undertaken to address perturbations in hemoglobin levels were appropriate was unknown; we thus assumed that practitioners were responding to the best of best of their ability to out-of-range hemoglobin values based on anemia management protocols and medical convention. 

In summary, we sought to determine whether and how hemoglobin variability has changed since the introduction of the revised PPS, and how hemoglobin variability is associated with outcomes. Not only did hemoglobin levels and ESA doses decrease, as expected, but even patients with persistently low hemoglobin levels received substantially less ESA in 2012 than in 2004. Transfusions appeared to be used more often than in 2004, especially in patients with persistently low hemoglobin levels, perhaps as a form of acute therapy. Low hemoglobin levels, rather than hemoglobin variability itself, appeared to be associated with the highest risk of mortality and MACE, suggesting that such patients may require the greatest scrutiny at the bedside. 

## Acknowledgment 

The authors thank Chronic Disease Research Group colleague Nan Booth, MSW, MPH, ELS, for manuscript editing. 

## Funding 

This work was supported by a research contract with Akebia Therapeutics, Inc. 

## Conflict of interest 

The analysis, interpretation, and reporting of these data are the responsibility of the authors. Dr. Gilbertson has provided consultation to DaVita Clinical Research. Ms. Hu and Mr. Peng report no conflicts of interest. Dr. Maroni is employed by Akebia therapeutics, Inc. Dr. Wetmore has served on advisory boards for Alexion Pharmaceuticals, Inc. 

**Table 1.** Patient characteristics overall and by hemoglobin variability group, 2004 and 2012 cohorts.

			Hemoglobin variability groups*											
	Overall		LL		II		HH		LI		IH		LH	
	2004	2012	2004	2012	2004	2012	2004	2012	2004	2012	2004	2012	2004	2012
Total n	159,720	200,728	2165	2200	9646	18,999	3750	10,552	29,222	48,029	50,680	60,525	64,257	60,423
Age, mean, yrs. (SD)	62.3 (15.2)	63.0 (14.7)	55.9 (15.9)	58.2 (15.7)	63.7 (14.8)	65.2 (14.2)	62.1 (15.4)	57.8 (13.7)	61.5 (15.2)	63.3 (14.8)	62.9 (14.9)	63.4 (14.6)	62.1 (15.3)	62.9 (15.0)
Age														
0 – 44	13.9	11.6	25.3	19.9	11.6	8.6	15.0	16.6	15.1	11.5	12.7	10.8	14.4	12.2
45 – 64	36	39.5	41.6	42.3	34.7	37.0	35.4	52.1	37.3	38.6	35.8	39.8	35.6	38.4
65 – 74	26.3	25.0	19.6	22.0	27.2	26.2	26.3	19.2	25.7	25.5	26.7	25.0	26.3	25.2
≥ 75	23.8	23.9	13.5	15.8	26.6	28.1	23.4	12.1	22.0	24.5	24.9	24.3	23.6	24.2
Sex														
Female	48.7	46.0	46.1	44.9	45.9	47.5	43.7	22.7	49.0	49.7	47.3	43.2	50.55	49.6
Male	51.3	54.0	54.0	55.1	54.1	52.5	56.3	77.3	51.0	50.3	52.7	56.8	49.44	50.4
Race														
White	53.4	53.1	48.5	45.8	58.5	54.9	51.3	53.8	54.7	51.6	53.4	55.0	52.36	52.0
Black	40.3	40.3	46.6	48.8	35.0	37.6	41.6	40.3	39.2	41.3	40.3	38.9	41.32	41.4
Other	6.3	6.6	4.9	5.4	6.5	7.5	7.1	5.9	6.2	7.0	6.3	6.1	6.32	6.6
Dialysis duration, mean, yrs. (SD)	4.1 (4.3)	5.6 (5.0)	4.9 (4.9)	5.9 (5.5)	4.2 (4.1)	5.4 (4.7)	3.9 (4.4)	7.1 (5.8)	4.4 (4.3)	5.6 (4.9)	4.0 (4.3)	5.5 (5.1)	4.0 (4.3)	5.4 (5.1)
Primary cause of ESRD														
Diabetes	42.8	44.6	35.1	35.9	43.4	46.5	42.4	36.2	42.7	45.3	42.9	44.7	43.0	45.2
Hypertension	29.8	29.9	27.3	27.0	28.4	30.1	30.4	30.3	28.5	29.3	30.5	30.4	30.0	29.8
Other	27.4	25.5	37.6	37.0	28.2	23.4	27.3	33.4	28.8	25.4	26.7	24.9	27.0	25.0
Hospitalization														
No	55.0	63.0	32.2	37.8	74.4	77.1	71.3	81.1	48.6	55.4	66.7	71.9	45.58	53.5
Yes	45.0	37.0	67.8	62.2	25.6	22.9	28.7	18.9	51.5	44.6	33.3	28.1	54.42	46.5
Total hospital days, mean, yrs. (SD)	5.0 (9.6)	4.1 (8.6)	12.3 (16.8)	10.9 (15.3)	1.9 (5.4)	1.9 (5.2)	2.1 (5.6)	1.4 (4.3)	6.7 (11.5)	5.6 (10.4)	2.7 (6.3)	2.5 (6.0)	6.4 (10.4)	5.6 (9.8)
Comorbidity														
ASHD	31.4	32.8	34.9	39.9	24.0	26.5	25.4	21.6	34.0	36.2	26.8	29.2	35.2	37.4
CHF	29.0	29.4	42.9	46.0	19.3	22.4	20.4	16.5	33.4	34.4	22.9	24.3	33.3	34.5
CVA/TIA	10.3	11.6	12.1	12.3	6.6	8.4	6.9	5.2	11.1	13.0	8.2	9.6	12.3	14.4
PVD	24.0	26.6	31.3	34.5	16.1	20.0	18.7	19.0	26.9	29.3	19.6	23.1	27.5	31.1
Other cardiac	21.5	19.6	35.8	35.8	14.6	13.9	14.0	10.6	25.7	23.9	16.1	15.0	24.8	23.6
COPD	12.8	16.0	18.0	25.3	8.0	11.4	9.3	9.6	15.0	18.5	9.8	13.4	14.9	18.8
GI bleeding	6.5	6.0	16.3	18.5	2.8	2.8	2.8	2.1	8.2	8.0	3.6	3.5	8.3	8.0
Liver disease	7.8	6.0	13.8	11.9	7.5	4.9	6.7	5.3	8.7	6.1	6.7	5.8	8.1	6.3
Dysrhythmia	19.6	23.9	27.4	34.0	15.2	19.4	15.3	17.2	22.3	27.1	16.2	21.0	21.8	26.5
Cancer	5.4	6.0	9.3	13.2	4.8	5.1	3.6	3.1	6.1	7.5	4.3	4.8	5.9	6.6
Diabetes	55.0	61.1	51.1	57.0	53.0	61.5	51.7	49.4	55.5	62.9	53.6	59.9	56.3	62.8

Unless otherwise indicated, data are percentages. LL = consistently low; LI = low-intermediate; LH = low-high; HH = consistently high; IH = intermediate-high; II = consistently intermediate; ASHD = atherosclerotic heart disease; CHF = congestive heart failure; COPD = chronic obstructive heart disease; CVA/TIA = cerebrovascular accident/transient ischemic attack; ESRD = end-stage renal disease; GI = gastrointestinal; PVD = peripheral vascular disease.
